# Cancer registry as external control data for regulatory submission in Japan

**DOI:** 10.1016/j.esmorw.2024.100072

**Published:** 2024-09-13

**Authors:** H. Bando, N. Okita, Y. Sakamoto, H. Sokuoka, Y. Nakamura, T. Hashimoto, T. Misumi, Y. Takeda, Y. Aoyagi, K. Mizuguchi, H.S. Okuma, N. Fuse, K. Yonemori, K. Nakamura, N. Yamamoto, T. Yoshino, A. Ohtsu

**Affiliations:** 1 Translational Research Support Office, Division of Drug and Diagnostic Development Promotion, Department for the Promotion of Drug and Diagnostic Development, National Cancer Center Hospital East, Kashiwa, Chiba; 2 Department of Gastroenterology and Gastrointestinal Oncology, National Cancer Center Hospital East, Kashiwa, Chiba; 3 Division of Data Science, Department for the Promotion of Drug and Diagnostic Development, National Cancer Center Hospital East, Kashiwa, Chiba; 4 Research Management Division, Clinical Research Support Office, National Cancer Center Hospital, Chuo-ku, Tokyo; 5 International Research Promotion Office, Division of Drug and Diagnostic Development Promotion, Department for the Promotion of Drug and Diagnostic Development, National Cancer Center Hospital East, Kashiwa, Chiba; 6 Information Technology Management Section, Clinical Research Management Division, Clinical Research Support Office, National Cancer Center Hospital East, Kashiwa, Chiba; 7 Department of Medical Information, National Cancer Center Hospital East, Kashiwa, Chiba; 8 Department of Medical Oncology; 9 Department of International Clinical Development, National Cancer Center Hospital, Chuo-ku, Tokyo; 10 Department of Clinical Research Support Office, National Cancer Center Hospital East, Kashiwa, Chiba; 11 Department of Experimental Therapeutics, National Cancer Center Hospital, Chuo-ku, Tokyo; 12 Clinical Research Support Office, National Cancer Center Hospital, Chuo-ku, Tokyo; 13 National Cancer Center Hospital East, Kashiwa, Chiba, Japan

**Keywords:** external control data, new drug application, real-world data, real-world evidence, data relevancy, data reliability

## Abstract

Through the Clinical Innovation Network, Japan’s regulatory authorities have enhanced the development of registries that utilize real-world data (RWD). The Ministry of Health, Labour and Welfare has issued guidelines, whereas the Pharmaceuticals and Medical Devices Agency has conducted consultations to manage and verify the integrity of these registries, thus improving the framework for the effective use of RWD. The use of cancer registry data as an external control group has been promoted by regulatory bodies and academic institutions. Given the aforementioned background, several high-quality cancer registries, such as the ‘SCRUM-Japan Registry’, ‘MASTER KEY project’, and ‘GALAXY registry’, have been established. The SCRUM-Japan Registry has been instrumental in achieving the world’s first regulatory approval for human epidermal growth factor receptor 2 (HER2)-positive colorectal cancer, demonstrating the value of regulatory-grade registries in managing rare molecular subtypes. However, the broader adoption of registry data for regulatory use in Japan remains limited, primarily owing to the lack of clear standards for using RWD/real-world evidence (RWE) for drug approval. This uncertainty has made pharmaceutical companies hesitant to use such data for regulatory submissions. This review aimed to elucidate the perspectives and related guidelines of the regulatory authorities concerning cancer registries in Japan. In response, the ‘REALISE study’ was initiated to define the ‘relevancy’ and ‘reliability’ of data necessary for new drug approvals and to develop methodologies to ensure data reliability retrospectively. The findings of this study will inform the creation of draft guidelines aimed at broadening the application of RWD/RWE throughout Japan.

## Highlights

• The use of cancer registry data is actively promoted by regulatory bodies in Japan.
• The SCRUM-Japan Registry plays a role in the first approval for HER2-positive colorectal cancer.
• The REALISE study defines the relevancy and reliability of data for new drug approvals.

## Introduction

According to the definition provided by the United States Food and Drug Administration (FDA), real-world data (RWD) encompass information related to patient health status and/or health care delivery that is routinely collected from a myriad of sources. These sources include, but are not limited to, electronic health records (EHRs), medical claims data, product or disease registries, and other means of informing about health status.^1^^,^^2^ Real-world evidence (RWE) refers to the clinical evidence regarding the use, potential benefits, or risks of a medical product derived from the analysis of RWD.^1^

In recent years, regulatory authorities worldwide have advocated the adoption of policies promoting the use of RWD. The utilization of RWD spans various domains, including basic research, planning of drug development, patient recruitment for clinical trials, regulatory submissions, and post-marketing surveillance. Pharmaceutical companies are increasingly utilizing RWD in response to increasing drug development costs, the issuance of various guidelines by regulatory authorities, and the growing body of use cases.^3^ A remarkable application of RWD/RWE is the creation of external control groups in clinical trials targeting rare diseases and molecular subtypes. While randomized controlled trials (RCTs) are unequivocally the gold standard for evidence generation, they pose significant challenges to these populations. Utilizing RWD/RWE as an external control group offers a methodology that can achieve regulatory approval for populations that are difficult to recruit for conventional randomized trials. Furthermore, RWD/RWE potentially reduces development costs, shortens development timelines, and alleviates ethical concerns. Conversely, regulatory authorities at the FDA and the European Medicines Agency (EMA) view selection bias and confounding as the most common issues related to external control groups.^4^ Ongoing scientific and practical discussions aim to elucidate the relevance and reliability of external control groups in a fit-for-purpose manner and to promote more widespread regulatory use of RWD and RWE.^5^

In Japan, the Clinical Innovation Network (CIN) was established under the 2016 Japan Revitalization Strategy to enhance the environment for efficient clinical development. The CIN has significantly advanced the development of registries for various disease types. In the field of oncology, multiple registries targeting rare molecular subtypes, rare cancers, and pediatric cancers have been developed. In addition, development of guidelines and initiation of consultations by regulatory authorities have actively promoted the utilization of these registries. In Japan, drug approval and insurance reimbursement are simultaneously conducted, and any expansion of indications requires an approval process. For expanding indications, including those for rare diseases, clinical trials are mandatory for regulatory submissions. When randomized clinical trials are not practical, the use of RWD is strongly considered. According to regulatory authorities, pharmaceutical companies, and clinical stakeholders, the development of well-qualified registries targeting rare molecular subtypes, rare cancers, and pediatric cancers is essential.

In this review, we aimed to elucidate the perspectives and related guidelines of the regulatory authorities concerning cancer registries in Japan. Additionally, we described the current status of cancer registry development, focusing on their application to various regulatory matters, including the use of external control data for regulatory submissions.

## Japan’s initiatives for integrating real-world data into regulatory use

In Japan, the establishment of the CIN was determined as a measure to enhance the environment for efficient clinical development in accordance with the Japan Revitalization Strategy of 2016. A conditional early approval system was introduced for pharmaceutical products in 2017. This system allows post-marketing surveys to be conducted using medical information databases and patient registries, indicating a broader utilization of these resources for various drug approval indications. Furthermore, the revision of the Good Post-marketing Study Practice Ministerial Ordinance, enforced in 2018, delineated the use of medical information databases for post-marketing surveillance of pharmaceutical products.

Since April 2019, the Pharmaceuticals and Medical Devices Agency (PMDA) has initiated the ‘Consultation for Development of Registry’ as a pilot project. Through this consultation, the PMDA offers guidance to registry holders, such as academic institutions, on planning concepts predicated on registry use, along with general principles for enhancing relevancy and ensuring the reliability of the registry. Additionally, the ‘Consultation for Pre-inspection on Registry Data Reliability’ provides advice, primarily to marketing authorization holders, on the assurance of registry reliability or verification of survey reliability before application submission. These initiatives have been fully operational since December 2020. Simultaneously, in March 2021, the Ministry of Health, Labour and Welfare (MHLW) issued additional guidelines entitled ‘Basic Principles on Utilization of Registry for Applications’ and ‘Points to Consider for Ensuring Reliability in the Utilization of Registry Data for Applications’. These guidelines were developed to encapsulate insights derived from cases where registries were used and foster their future applications^6^^,^^7^ (Table 1).

**Table 1:** Consultation contacts and guidelines related to the registry

Consultations for registry utilization by the PMDA	
Consultation for Development of Registry	The consultation addresses the use of registries that may be relevant for the submission and approval or reassessment of pharmaceuticals and regenerative medicine-related products. It particularly highlights registries maintained by academic institutions, such as universities, research organizations, and scholarly societies. The PMDA provides guidance on how to plan for the use of these registries. It emphasizes the development of strategies to improve their quality and to ensure their reliability for use in regulatory evaluations.
Consultation for Pre-inspection on Registry Data Reliability	The consultation addresses the utilization of registries in the approval or re-evaluation processes for pharmaceuticals and regenerative medicine products. It provides opinions of the PMDA on the principles necessary to ensure the reliability of these registries before the commencement of any related research or studies. Furthermore, it involves conducting checks on the reliability of such research or studies before the submission of applications, thereby ensuring compliance with established reliability standards.
Issuance of guidelines by the MHLW	
Basic Principles on Utilization of Registry for Applications	This guideline is designed to establish fundamental principles for the use of registry data in applications, irrespective of the data source’s country of origin. It outlines basic principles applicable to scenarios where data are sourced exclusively from registries or augmented through linkage with information from other data sources.
Points to Consider for Ensuring the Reliability in Utilization of Registry Data for Applications	This guideline encompasses both the creation of new registries and the utilization of existing registries that have accumulated data over time.

MHLW, Ministry of Health, Labour and Welfare; PMDA, Pharmaceuticals and Medical Devices Agency.

The ‘Guidelines for Clinical Evaluation Methods for Antineoplastic Drugs’ were also simultaneously revised.^8^ The guideline stated that “For drugs targeting rare cancers or rare disease subtypes with a small number of patients, it is difficult to conduct a comparative confirmatory study. Therefore, they may be evaluated in a single-arm phase II study. In that case, explanations of clinical usefulness in comparison with the historical data, such as disease registries, can be considered”. This approach has led health care authorities to establish a framework for the utilization of RWD and RWE for drug approval and post-marketing surveillance activities.

## Representative cancer registries as external control data for regulatory submission in japan

To improve precision medicine in Japan, the National Cancer Center Hospital East (NCCHE) and the National Cancer Center Hospital have established a comprehensive cancer genome screening system and cancer registries of rare cancers and molecular subtypes (Table 2).

**Table 2:** Comparison of the components of three oncology-specific registries in Japan

	SCRUM-Japan Registry	MASTER KEY project	GALAXY registry
Cancer type	Unresectable solid tumors	Rare cancers and hematological malignancies	Resectable colorectal cancer
Type of registry	This study aims to prospectively collect treatment information and treatment efficacy data from patients with unresectable solid tumors who have specific genetic abnormalities detected in research related to SCRUM-Japan. The goal is to establish external control group data for clinical trials and other research purposes.	This study aims to prospectively collect data on the biological background, clinical progression, and prognosis of patients with incurable progressive lesions, including rare cancers, cancers of unknown primary origin, and rare subtypes of common cancers, all characterized by specific biomarkers.	The study is a registry targeting patients with colon and rectal cancer who are scheduled for curative surgery. Whole-exome sequencing (WES) of tumor samples is conducted to identify genetic changes in both tumor tissue and blood samples, and the clinical progression is examined as the natural history.
Use for registry-based studies and previous publications	Utilization of external control groups for the regulatory approval of pertuzumab and trastuzumab combination therapy for HER2-positive unresectable colorectal cancer in Japan https://www.sciencedirect.com/science/article/pii/S1533002822001074?via%3Dihub	The registry is not yet utilized for publication but is available as a prospective registry platform. A paper introducing the registry and detailing the data collection status has been published as follows: https://www.ncbi.nlm.nih.gov/pmc/articles/PMC7484913/	A paper based on collected data regarding the efficacy of circulating tumor DNA (ctDNA) and adjuvant therapy in colorectal cancer has been published. https://doi.org/10.1038/s41591-022-02115-4
Geographical and organizational setting	Geographical area: Japan Organizational setting: National Cancer Center Hospital East	Geographical area: Japan Organizational setting: National Cancer Center Hospital	Geographical area: Japan, Taiwan Organizational setting: National Cancer Center Hospital East
Duration	Data collection start date: 3 July 2017 Projected data collection end date: 31 March 2026	Data collection start date: May 2017 The projected data collection end date has not been determined.	Cases will be registered from 8 May 2020 to 31 March 2024. The clinical course of the registered cases will be monitored as part of their natural history until 31 March 2031.
Size (as of March 2024)	544 cases	3947 cases	6061 cases
Inclusion and exclusion criteria	Inclusion criteria Subjects must meet all of the following inclusion criteria (1-4) to be eligible: 1) Unresectable solid malignancies. 2) Performance of comprehensive genomic profiling through next-generation sequencing or SCRUM-Japan-related research. 3) Identification of a specific genetic alteration. 4) Minimum age of 20 years at the time of registration. Exclusion criteria Any condition that, in the opinion of the investigator, would preclude participation in the research.	Inclusion criteria 1) Age of 0 years or more at registration. 2) Histopathological diagnosis of one of the cancers defined in ‘3.1. Definition of Rare Cancers’, cancers of unknown primary origin, or a rare histological subtype of common cancer as specified in ‘3.2. Rare Histological Subtypes of Common Cancers Targeted in This Study’. 3) Incurable progressive (metastatic and/or unresectable) lesions. 4) Performance of next-generation sequencing or molecular testing (immunohistochemistry, FISH, etc.), request for such testing, or provision of oral or written consent for such testing. 5) Provision of written informed consent for participation in the study. For patients under 18 years of age, consent must be obtained from a legally authorized representative.	Inclusion criteria Patients must meet all of the following criteria. Additionally, patients who experience recurrence after initial registration and are eligible for a second curative surgical resection can be re-registered. 1) Histopathological diagnosis of adenocarcinoma. 2) Primary tumor site being the colon (cecum, colon, sigmoid colon) or rectum (excluding appendix and anal canal cancer). 3) <Cohort A> Colon cancer (including RS) with clinical stage II or III according to UICC eight edition, scheduled for curative resection. <Cohort B> Rectal cancer (excluding RS) with clinical stage II or III according to UICC eight edition, scheduled for curative resection. However, cases with lateral lymph node metastasis are eligible for registration. <Cohort C> Colon or rectal cancer with clinical stage IV or recurrence (M1) according to UICC eight edition, scheduled for curative resection. <Cohort D> pT1 colon or rectal cancer after local resection scheduled for additional colorectal resection with lymph node dissection due to non-curative factors. <Cohort E> Patients with stage IIB, IIC, or III colorectal cancer according to UICC 8th edition who have undergone curative resection within 48 weeks before enrollment. 4) Age of ≥20 years at the time of obtaining consent. 5) Eastern Cooperative Oncology Group performance status of 0 or 1. 6) Provision of written informed consent for participation in this study. Exclusion criteria 1) Presence of two or more synchronous primary colorectal cancers (multiple cancers). 2) Presence of active multiple cancers. 3) <For cohort A and cohort D registration> History of surgery, chemotherapy, immunotherapy, or radiation therapy within 6 months before registration. 4) Pregnant or breastfeeding women. 5) Presence of severe comorbidities. 6) Positive for HBs antigen or HCV antibodies. 7) Positive for HIV antibodies. 8) Presence of active COVID-19 infection. 9) Unsuitable for participation as determined by the attending physician.
Follow-up	15.3 months (413 cases)	The average observation period is 526 days. Follow-up surveys are conducted approximately every 6 months. If a patient is transferred to another hospital, their outcome is confirmed via phone or other means.	Data on the clinical course will be prospectively collected as part of the natural history until 31 March 2031.
Confounders	Because of the high number of cases registered after the completion of standard treatments, there is a noted trend towards prolonged OS times. Although controlling for this trend is difficult, users should be informed of it to account for this factor during analysis and at the time of forming conclusions.	As information is collected to understand the natural history, no confounding factors are excluded or adjusted during the data collection phase. Adjustments such as stratification will be carried out during the statistical analysis phase, depending on the specific use of the registry.	Data are collected as part of the natural history; therefore, no adjustments for confounding factors are made during the data collection phase. Adjustments such as stratification will be conducted during the statistical analysis phase, according to the particular application of the registry.
Registry aims and methodology	The study is conducted according to the Study Protocol (Version 3.6).	The study is conducted according to the Study Protocol (eight edition, effective as of 2 August 2023). https://www.ncc.go.jp/jp/ncch/masterkeyproject/outline/overview/master_key_project_overview.html	The study protocol provides a detailed overview of the objectives, target population, endpoints, and research methodology.
Governance	This research has been reviewed and approved by the Institutional Review Board of the National Cancer Center Japan, based on the ‘Ethical Guidelines for Life Sciences and Medical Research Involving Human Subjects’. After obtaining approval, the study received implementation permission from the President of the National Cancer Center Japan and is being conducted under this authorization.	The study follows the Declaration of Helsinki and the Ethical Guidelines for Life Sciences and Medical Research Involving Human Subjects. It has been reviewed by the Research Ethics Review Committee to ensure compliance, ethical considerations, and scientific validity. Approval from the committee and the head of the medical institution will be obtained before implementation.	The GALAXY registry is evaluated by the Institutional Review Board (IRB) for compliance with the Declaration of Helsinki and the ‘Ethical Guidelines for Medical and Health Research Involving Human Subjects’. The IRB ensures that the registry meets these guidelines and upholds ethical and scientific standards. Upon IRB approval, the registry is carried out with the permission of the head of the medical institution.
Informed consent	Consent has been obtained in a documented consent form describing secondary use, including use by industry.	Consent has been obtained in a documented consent form describing secondary use, including use by industry.	Consent has been obtained in a documented consent form describing secondary use, including use by industry.
Data dictionary	Modifications to the electric data capture (EDC) system items can be made as necessary. When such modifications are required, the EDC vendor will undertake system adjustments based on the specifications provided by the data holder.	Information is collected from medical institutions through an EDC system. The data items are defined in a requirements specification document during the EDC setup. Data can be processed for specific purposes as needed.	Data collection from medical institutions is conducted through the EDC system. The items for data collection are defined in a requirements specification during EDC setup, and the data can be processed for particular purposes as required.
Minimum dataset	In collaboration with the users, we verify the criteria for selecting eligible cases for data use and compile a list of these cases. Using this list, we extract data for the eligible cases from the EDC system and designate this as raw data.	Following established procedures and in consultation with users, the conditions for the cases to be included in the data are confirmed and a list of target cases is created. Stakeholders can view an overview of the registered data through the utilization system and, upon request, data can be exported from the EDC. Based on the target case list, necessary cases are extracted and treated as raw data.	In accordance with the procedures and in collaboration with the users, the criteria for eligible cases for data use are confirmed and a list of eligible cases is created. Data are extracted from the EDC system, and the required cases are identified based on the eligible case list and designated as raw data.
Standard definitions, terminology, and specifications	The data are stored in accordance with the database definitions configured within the EDC system. The EDC system is implemented based on the security specifications summarized below. Furthermore, data exported from the EDC system can be formatted in compliance with the CDISC Study Data Tabulation Model standards.	The raw data can be processed into datasets according to definitions such as CDISC, as requested by users.	The raw data can be transformed into datasets according to CDISC or other definitions, as requested by the users.
Data collection	Data are collected using the EDC system. Users from collaborating research institutions (site users) are issued accounts with data entry privileges after completing EDC training. Site users will then review their institution’s medical records and input the required information into the EDC system. The data holder will export only the necessary information from the EDC system and provide it to the users.	Data are collected using the EDC system. Medical institution users are granted account access with data entry permissions after completing EDC training. Facility users review their institution’s medical records and enter the necessary information into the EDC according to the input manual. Data holders export data from the EDC and provide users with only the necessary information.	Data are collected using the EDC system. Users from medical institutions receive accounts with data entry privileges after completing EDC training. These users review their institution’s medical records and input the required information into the EDC according to the data entry manual. The data holder exports data from the EDC and provides only the necessary information to the users.
Quality assurance	The data collected via the EDC system are cleaned at the data center. Any errors detected during the data cleaning process are followed up by issuing queries to the site personnel for correction. This process is repeated until all errors are resolved. Additionally, onsite monitoring may be conducted as necessary to verify that the information from medical records is accurately transcribed into the EDC system.	Trained facility clinical research coordinators or dispatched data managers enter data into the EDC. Quality assurance is ensured through sampling source data verification to check the consistency of medical record data and by conducting central monitoring using the data entered into the EDC.	Depending on the specific purposes of the registry, auditors will conduct reviews at medical institutions by directly inspecting and cross-referencing source documents. This process will ensure that clinical research is being appropriately conducted, that the reliability of the data is maintained, and that the research implementation framework is suitably constructed and managed.
Data cleaning	An annual data update is carried out for all cases, and additional data cleaning is conducted for cases selected for specific utilization.	Data collected in the EDC are subjected to data cleaning at the data center. Errors identified during data cleaning are addressed by issuing queries to facility personnel, requesting corrections. This process is repeated until all errors are resolved.	Data collected via the EDC system are subject to data cleaning at the data center. Any errors identified during data cleaning are managed by issuing queries to the site personnel, requesting corrections. This process is repeated until all errors are resolved.
Missing data	At present, there is no defined method for handling or presenting missing data.	Progress and adherence to the study protocol are monitored using the data entered into the EDC, with monitoring reports generated twice a year. These reports also track data collection status by item.	Annual monitoring reports are generated using data entered into the EDC system and other relevant sources to track progress and adherence to the study protocol. These reports also provide insights into the status of data collection by item.
Financing	This study is supported by research grants from the National Cancer Center Hospital East and research funds obtained through utilization by pharmaceutical companies.	Necessary expenses are invoiced in installments based on contracts concluded with collaborating companies, which serve as the funding source. The registry is financially maintained within this budget.	This study is supported by research grants from the National Cancer Center Hospital East.
Protection, security, and safeguards	To prevent unauthorized access to the EDC system, SSL encryption, antivirus protection, and firewall measures are in place. Additionally, identity authentication (ID/password) is implemented to prevent impersonation. Daily data backups are carried out to protect against data loss due to EDC server failures, and data from the past 30 days are retained.	Measures against unauthorized access to the EDC include SSL encryption, antivirus protection, and firewall installation. Additionally, identity verification (ID/password) is implemented to prevent impersonation.	Users must comply with the ‘Ethical Guidelines for Life Sciences and Medical Research Involving Human Subjects’, the ‘Act on the Protection of Personal Information’, the ‘Act on the Protection of Personal Information Held by Incorporated Administrative Agencies’, the ‘Guidelines for the Safe Management of Medical Information Systems’, and all relevant laws and regulations, including amendments, when using the data provided by the holder. Additionally, users must adhere to the laws and guidelines of their own countries and local jurisdictions (or their equivalents) when utilizing the data. Users are also required to observe the following key principles regarding the data provided by the registry holder and any secondary research or data utilization derived from these data: • Restriction of data users (limited to the principal investigator, co-investigators, and authorized personnel from contracted external organizations as specified in the application). • Explicit statement of intended use. • Prohibition of use for any purposes other than those specified in the application. • Prohibition of sale. • Prohibition of use for weapon development or military purposes. • Prohibition of identification of individuals. • Prohibition of redistribution.
Consultation for Development of Registry	Yes	Yes	Yes
Consultation for Pre-inspection on Registry Data Reliability	Not yet	Not yet	Yes
Evaluation of efficacy	Imaging evaluations were carried out at 8 weeks ± 2 weeks. Efficacies were evaluated using the RECIST, and evaluations of efficacies, such as RR, PFS, and OS, can be carried out.	Imaging evaluation is recommended to be conducted every 8-12 weeks. The data are collected as per the normal course of clinical practice.	Based on the guidelines for colorectal cancer treatment, the plan involves conducting tumor marker assessments every 3 months and imaging evaluations through CT scans every 6 months for up to 5 years post-surgery.
Genome data	Next-generation sequencing data are attached in all cases.	Next-generation sequencing data are collected in 58% of cases.	Data on *RAS* and *BRAF* mutation status, microsatellite instability, and WES results are attached in all cases.
Legal compliance for regulatory submissions	Data can be used for regulatory approval.	Data can be used for regulatory approval.	Data can be used for regulatory approval.

COVID, coronavirus disease; CT, computed tomography; HBs, hepatitis B surface; HCV, hepatitis C virus; OS, overall survival; PFS, progression-free survival; RR, response rate; RS, rectosigmoid; SSL, Secure Sockets Layer; UICC, Union Internationale Contre le Cancer.

### SCRUM-Japan and SCRUM-Japan Registry

In the era of a data-driven society, the NCCHE has been actively accumulating high-quality clinical data combined with genome and various types of omics data. SCRUM-Japan is the largest nationwide cancer genome screening project in Japan based on industry–academia collaboration. In 2015, SCRUM-Japan was launched by combining the ‘LC-SCRUM-Japan’ and ‘GI-SCREEN-Japan’ (UMIN000016343 and UMIN000016344) platforms, as Japan’s first industry–academia nationwide cancer genome screening project. Since then, we have launched various studies, including the ‘GOZILA study (UMIN000029315)’ in 2018, ‘MONSTAR-SCREEN-1 project’ (UMIN000036749) in 2019, and ‘MONSTAR-SCREEN-2 project’ (UMIN000043899) in May 2021.^9^

Numerous investigator-initiated studies have been conducted within the framework of the SCRUM-Japan screening platform and its associated research endeavors, with the aim of assessing the efficacy of targeted therapeutic agents for rare subtypes.^9^^,^^10^ In clinical trials targeting these rare molecular subtypes, which represent 1%-5% of the total population, achieving a sufficient sample size for RCTs presents significant challenges owing to cost and time constraints. To address these challenges, we established the SCRUM-Japan Registry, a regulatory-grade registry that defines prospectively constructed registries designed for regulatory submissions and focuses on the collection of high-quality clinical data based on its standard operating procedures (SOPs). Within the SCRUM-Japan Registry, we proactively gather clinical data on standard therapies for patients with rare genetic alterations, in anticipation of new drug approval applications for mutations, such as *BRAF*, *ERBB2*, *MET*, *BRCA*, *FGFR*, and *NFE2L2*.^11^ The initiation of the SCRUM-Japan Registry was under the guidance of the Japan Agency for Medical Research and Development (AMED) Project Promoting Clinical Trials for Development of New Drugs (Ohtsu Group, AMED) in 2016, with the commencement of patient registration in November 2017.

Patients exhibiting specific genetic alterations identified through SCRUM-Japan and associated studies were enrolled with informed consent from individual patients. Following enrollment, the effectiveness of standard therapy in clinical settings was assessed using computed tomography (CT) within 6 weeks before the commencement of each treatment regimen and subsequently every 8 ± 2 weeks in accordance with the registry’s protocol. Key metrics for standard treatments, such as response rate, disease control rate, progression-free survival, duration of response, and time to treatment failure, were evaluated using prospectively collected CT images. Additionally, overall survival (OS) was systematically monitored in accordance with the registry protocol guidelines. Clinical data accumulated before enrollment in the SCRUM-Japan Registry were also retrospectively gathered.^12^

The collected data are entered into a dedicated electronic data capture (EDC) system by an educated local data manager, site investigator, or clinical research coordinator at each study site, all of whom are trained in the data entry rules. The EDC system is managed using an audit trail to ensure future usability. To maintain clinical data consistency, a manual for data entry and editing checks within the EDC are utilized. Data cleaning is carried out, and queries are issued when inconsistencies are detected. Moreover, both central and onsite monitoring are implemented to ensure data reliability. Based on the outcomes of the central monitoring, we request corrective actions from the principal investigators at sites exhibiting higher rates of deviation to enhance operations. Audits are conducted for both the study sites and registry holders.^12^

Given the imperative for the SCRUM-Japan Registry to ensure the relevance and reliability of new drug applications, we sought the PMDA ‘Consultation for Utilization of Registries’ in July 2019. During this consultation, the procedures of the SCRUM-Japan Registry management system and the validity of data utilization for external control were discussed. Recommendations from the PMDA were integrated into the procedures and management system of the SCRUM-Japan Registry. Furthermore, in accordance with the ‘Points to Consider for Ensuring the Reliability of Registry Data for Applications’, we reviewed and refined the standard procedures and documentation for the organization, computer system, data extraction, and dataset creation of the SCRUM-Japan Registry.^12^

### MASTER KEY project

The development of treatments for rare cancers, defined by an annual incidence of <6 cases per 100 000 individuals,^13^ has faced substantial delays. These delays stem from various challenges, including the scarcity of natural history data, difficulties in achieving accurate diagnoses, obstacles in accruing sufficient patient numbers for clinical trials, and the complexities involved in conducting randomized trials. To address these challenges, the MASTER KEY project was initiated as a platform trial. This initiative combines a prospective registry study with multiple clinical trials, focusing specifically on rare cancers. It encompasses both solid tumors and hematological malignancies, leveraging an industry–academia collaboration that commenced in May 2017.^14^ As of April 2024, the project has expanded to involve seven clinical sites in partnership with 12 pharmaceutical companies.^15^ This collaborative effort also includes engaging with patient associations to foster a comprehensive approach to tackling the unique challenges presented by rare cancer research and treatment development.

The core aim of this study is to collect detailed data on advanced rare cancers, with a focus on biomarkers, patient demographics, and prognostic indicators. The MASTER KEY registry recommends conducting CT evaluation every 8-12 weeks and collecting patient data as per the normal course of clinical practice or through clinical trial participation. Similar to the methodology of SCRUM-Japan, the MASTER KEY registry uses an EDC system for data entry managed by trained investigators or clinical research coordinators at each site. In addition, data managers from the central office may assist in data collection at various sites. Procedures for data entry and internal EDC checks are carried out to maintain consistency. Ensuring data reliability involves regular data cleaning and central and onsite monitoring based on risk-based principles, with additional checks carried out as required for quality assurance. In November 2019, the MASTER KEY registry was evaluated in a ‘Consultation for Utilization of Registries’ with the PMDA,^6^ which affirmed the adequacy of its data reliability measures.

The MASTER KEY project plays a pivotal role in advancing treatment for rare cancers, primarily through its clinical trial component, which conducts single-arm phase II trials targeting rare cancer types or those defined by specific biomarkers. Since March 2024, this initiative has supported 16 investigator-initiated and 14 industry-sponsored clinical trials.^15^ With complete patient enrollment in several studies, pharmaceutical companies are now considering regulatory submissions based on these results.

The MASTER KEY project prioritizes collaboration among industry, academia, and rare cancer patients while maintaining regular communication with patient associations. An annual ‘Rare Cancers Community Open Day’ event promotes clinical trial awareness and participation. Furthermore, the project partners submitted a joint letter to the MHLW highlighting the challenges in developing companion diagnostics (CDx) for rare cancers. This effort led to the issuance of a new notification by the MHLW notifying the relaxation of regulations regarding CDx development upon drug approval in investigator-initiated trials for rare cancers and showcasing the effects of collaborative advocacy in addressing the unique needs of rare cancer research and treatment development.^16^

### GALAXY registry

Adjuvant chemotherapy reduces the risk of tumor recurrence and improves the survival of patients with resected colorectal cancer. The potential utility of circulating tumor DNA (ctDNA) before and after surgery has been reported for various solid tumors.^17^ We initiated a new type of adaptive platform trial to evaluate the clinical benefits of ctDNA analysis and refine precision adjuvant therapy for resectable colorectal cancer (CRC), named CIRCULATE-Japan.^18^

The GALAXY registry is a prospectively conducted large-scale registry designed to monitor ctDNA in patients with clinical stages II-IV or recurrent CRC who can undergo complete surgical resection starting in 2020. The target number of enrolled patients is 6300 from 152 participating institutions. The registration period lasts until March 2024, with plans for long-term prognostic tracking until March 2031. Clinical information on patient background, disease-free survival, and OS was prospectively collected from the GALAXY registry. Additionally, data on *RAS* and *BRAF* mutation status, microsatellite instability, and whole-exome sequencing results were collected for all cases. Comprehensive and documented consent was obtained from the patients for the secondary use of the samples and information in applications related to manufacturing and marketing approval. Furthermore, to enhance the quality and ensure the reliability of the registry from the initial registration phase, we received the ‘Consultation for Utilization of Registries’ in April 2021. In this consultation, we received general guidance on the procedures for the operation and management of computer systems, response to incidents, management of data entry personnel, quality control and assurance of data, and preservation of records.

Data from the GALAXY registry were used as external data for the regulatory submissions. One future use case is the external control data for a multicenter phase II clinical trial to evaluate the efficacy and safety of perioperative chemotherapy with the combination of encorafenib, binimetinib, and cetuximab in patients with *BRAF* V600E-mutated CRC with resectable metastasis (EPOC2101, NEXUS trial, jRCT2031220025).^19^ The other use case is the external control data for a multicenter phase II clinical trial to evaluate the efficacy and safety of nivolumab in patients with mismatch repair-deficient resectable rectal cancer (EPOC2201, VOLTAGE-2 study, jRCT2031220484).^20^ Before starting clinical trials, we submitted the trial design to the PMDA, and the use of registry data has reached an agreement. In discussions with the PMDA, we were encouraged to seek a ‘Consultation for Pre-inspection on Registry Data Reliability’, which we subsequently undertook in May 2022. During this consultation, we were advised to revise the study procedure, strengthen the central monitoring, and proceed with risk-based source data verification for cases planned for use as external control groups.

### Use of SCRUM-Japan Registry: experience of the TRIUMPH study

The approval of pertuzumab and trastuzumab for human epidermal growth factor receptor 2 (HER2)-positive metastatic CRC (mCRC), based on the results of the TRIUMPH study—an investigator-initiated phase II trial conducted by the NCCHE (EPOC1602, UMIN000027887)—along with external control data from the SCRUM-Japan Registry, represents the first significant application of the SCRUM-Japan Registry.^21^ The TRIUMPH study evaluated the efficacy and safety of pertuzumab and trastuzumab as salvage-line therapies for patients with HER2-positive and *RAS* wild-type mCRC. Based on the results of the phase II TRIUMPH study and data extracted from the SCRUM-Japan Registry, the pharmaceutical company submitted an application for pertuzumab and trastuzumab administration for HER2-positive mCRC in April 2021.

The pharmaceutical company submitted case data from the TRIUMPH study (*n* = 30), the SCRUM-Japan Registry (*n* = 14), and the MyPathway trial^22^ (*n* = 57) along with case data registered in the real-world cancer genomic information database of Flatiron and Foundation Medicine^23^ (data on cases that received treatments selected by the primary care physician) (*n* = 18) to the PMDA. As the ‘Basic Principles on Utilization of Registry for Applications’ was issued after the start of construction of the SCRUM-Japan Registry (in March 2021), we reviewed the systems of our registry in terms of data quality and reliability. For ‘GCP On-site Inspection and Document-based Conformity Inspection’ conducted for the TRIUMPH study, the SOPs and documentation were revised. Specifically, we have added descriptions of the vendor assessment for computer systems, SOPs, monitoring procedures, and implementation records, as well as data extraction procedures, based on the ‘Basic Principles on Utilization of Registry for Applications’. Pertuzumab and trastuzumab were approved as expanded indications on 28 March 2022.^12^ To the best of our knowledge, this is the first regulatory approval for HER2-positive CRC worldwide and represents remarkable success in obtaining regulatory approval utilizing regulatory-grade registries for rare molecular subtypes.

According to the review report from the PMDA, 6 out of 14 cases from the SCRUM-Japan Registry were classified as ‘evaluation material’; this meant that each patient’s data were reviewed as control data for the TRIUMPH study. This classification followed the acquisition of informed consent and the prospective collection of clinical data. However, the remaining data, including those for 8 out of 14 cases from the SCRUM-Japan Registry, were classified as ‘reference material’; this meant that each patient’s data were not reviewed. For the eight cases classified as ‘reference material’, the evaluation intervals were not standardized because of the retrospective nature of the data.^24^ The data from Flatiron and Foundation Medicine included only OS data of patients treated with physician-selected therapies, unlike the regulatory-grade registry data from the SCRUM-Japan Registry.^12^

In the TRIUMPH study, the primary endpoint was met with a confirmed overall response rate of 30% for 27 tissue-positive patients and 28% for 25 ctDNA-positive patients.^21^ Moreover, the effectiveness of standard therapy for six HER2-positive and RAS wild-type patients treated with fluorouracil, oxaliplatin, irinotecan, anti-epidermal growth factor receptor antibody, and bevacizumab was extracted from the SCRUM-Japan Registry as ‘evaluation material’. The majority of the patients had received salvage treatment with trifluridine/tipiracil ± bevacizumab. Remarkably, none of the five patients who could be assessed by RECIST v 1.1 showed a response (0/5). The PMDA concluded that although the information provides useful insights into the efficacy of pertuzumab plus trastuzumab, the extremely limited number of patients studied and the potential bias due to variations in patient background factors limit the ability to directly compare the data from the SCRUM-Japan Registry with those from the TRIUMPH study.^24^

### The next SCRUM-Japan project including various types of solid tumors and hematological malignancies

Starting in May 2024, SCRUM-Japan will embark on its latest project, ‘MONSTAR-SCREEN-3’, which will extensively concentrate on both solid tumors and hematological malignancies. This study aims to elucidate the molecular pathology and spatiotemporal heterogeneity of these disorders by conducting longitudinal molecular profiling, including spatial transcriptomics. The study will include three distinct patient cohorts: cohort A will comprise patients with unresectable advanced solid tumors eligible for palliative chemotherapy, cohort B will include patients with advanced solid tumors amenable to potentially curative surgery or radical radiotherapy, and cohort C will include those diagnosed with hematological malignancies scheduled for chemotherapy.

For patients in cohorts A and C, comprehensive molecular profiling efforts will be made to identify molecular signatures that can serve as critical therapeutic targets. Similarly, patients in cohorts B and C will undergo molecular profiling tailored to their condition, utilizing whole genome sequencing to assess ctDNA for evaluating molecular residual disease.^17^ Additionally, the study will explore image profiling through the digitalization of pathology specimens and radiodiagnostic imaging. By applying artificial intelligence to analyze combined molecular and imaging data, along with clinicopathological parameters and patient outcomes, this project aims to develop biomarkers that accurately reflect the molecular biology of malignant tumors.

The study protocol carefully specifies the schedule for imaging assessments to ensure the systematic collection of clinical data, with the intention of integrating this information into a registry for future regulatory submissions. To enhance the reliability of the findings, the research methodology will include the creation of EDC systems, thorough input manuals, and explicit procedural instructions. In the near future, we should plan consultations to enhance the relevancy and reliability through the ‘Consultation for Development of Registry’.

## Development to extract the data with sufficient relevancy and reliability

When we attempt the utilization of RWD for the application of regulatory approval based on the fitness-for-purpose, the ‘relevance’ and ‘reliability’ of data should be considered.^25^^,^^26^ The ‘relevance’ of the data includes the availability of key data elements (exposure, outcome, and covariate), representativeness, sufficient subjects, and longitudinal data. The ‘reliability’ of data includes accuracy, completeness, provenance, and traceability of data processing.^25^^,^^26^ To broaden the application of RWD in regulatory contexts, ranging from actively compiled disease registries to standard databases and EHRs filled with clinical data, it is crucial to develop methods for selecting cases that meet the relevant criteria and to establish methodologies that retrospectively guarantee reliability. Recently, we have launched the ‘REALISE study’ to investigate these considerations through the use of diverse databases gathered by SCRUM-Japan, thereby possibly enhancing the further exploitation of RWD (UMIN000053533).^27^ This study aims to compare the ‘relevance’ and ‘reliability’ of four major databases: the ARCAD global database, SCRUM-Japan Registry, SCRUM-Japan observational study, and Flatiron Health RWD. Because the primary purpose of the four databases is to provide supplemental data for regulatory submissions, we will evaluate the ‘relevance’ of these databases based on their potential future regulatory use. For ‘reliability’, we will compare the collected data points and the systems for data quality control and quality assurance (Table 3).^27^ We will review the database using the checklists and management sheets issued by the PMDA.^28^ In addition, the REQueST tool from the EMA, NICE’s DATASAT, and the Structured Process to Identify Fit-For-Purpose Data (SPIFD) tool^29^ will be referred to as needed. We will statistically assess the differences and similarities of the four databases. This study will also focus on developing methods to effectively extract relevant data from the SCRUM-Japan observational study. If the RWD/RWE does not meet the reliability standards necessary for regulatory approval, we will explore methods to improve the reliability of the SCRUM-Japan observational study to meet these requirements. The findings will be submitted to the PMDA’s ‘Consultation for Development of Registry’ to discuss standard methodologies. Furthermore, the processes and outcomes of the REALISE study will be documented from the perspectives of ‘database construction’, ‘data analysis’, and ‘outcome evaluation’, culminating in the publication of ‘draft guidelines’.^27^

**Table 3:** The list of databases for the REALISE study^27^

	ARCAD global database	SCRUM-Japan Registry	SCRUM-Japan observational study	Flatiron health real-world data study
Cancer type	Colorectal cancer	Solid tumors	Solid tumors	Gastrointestinal cancers (real-word data for breast cancer, with lung cancer and hematological malignancies under development)
Sample size (as of January 2024)	ARCAD global database: 45 224 cases from 63 trials ARCAD Asia database: 4218 cases from 13 trials	546 cases	Total: 14 325 cases GI-SCREEN 2013-01-CRC: 3641 cases GI-SCREEN 2015–01-Non-CRC: 2952 cases MONSTAR-SCREEN: 2224 cases GOZILA study: 5508 cases	650 cases (Japan only)
Number of participating sites	ARCAD is the database project constructing three data centers.	72 sites	Total: 31 sites GI-SCREEN 2013-01-CRC: 26 sites GI-SCREEN 2015–01-Non-CRC: 24 sites MONSTAR-SCREEN: 31 sites GOZILA study: 31 sites	One site (on track to grow to more than five partners within 2024 and building multi-site data products based on that network. Continuing to grow Japan network in 2025+ as needed to ensure value and representativeness of datasets.)
Number of collected data points	Clinical characteristics: 72 Treatment data: 90 Efficacies: 17	Clinical characteristics: 39 Treatment data: 16 Efficacies: 12	Clinical characteristics GI-SCREEN 2013-01-CRC: 11 GI-SCREEN 2015–01-Non-CRC: 15 MONSTAR-SCREEN: 21 GOZILA study: 16 Treatment data GI-SCREEN 2013-01-CRC: 7 GI-SCREEN 2015–01-Non-CRC: 7 MONSTAR-SCREEN: 17 GOZILA study: 9 Efficacies GI-SCREEN 2013-01-CRC: 2 GI-SCREEN 2015–01-Non-CRC: 2 MONSTAR-SCREEN: 4 GOZILA study: 4	Clinical characteristics: 66 Treatment data: 19 standard data elements per drug delivered, as well as 13 additional standard data elements for radiotherapy and surgery episodes. These details are documented for every line of therapy captured throughout the patient journey. Efficacies: currently one, with additional clinical outcomes and endpoints being added in 2024 and beyond.
Data quality and reliability	Data from prospective randomized studies conducted for drug approval. Onsite and central monitoring, source document verification (SDV), and audits have been already conducted in several studies.	Efficacy data were prospectively collected. Onsite and central monitoring, SDV, and audits have been conducted for regulatory use.	Data were collected as an observational study. Data were cleaned annually.	Structured and unstructured data were collected from electronic health record. Data will be updated every 3 months. Procedures, including abstraction procedures, education for abstractors, and automatic verification on the system are carried out to ensure reliability.
Evaluation of efficacy	Imaging evaluations (computed tomography scan) were carried out every 6-8 weeks. Efficacies were evaluated using the Response Evaluation Criteria in Solid Tumors (RECIST), allowing evaluations of efficacies, such as response rate (RR), progression-free survival (PFS), and overall survival (OS), at the patient level.	Imaging evaluations were carried out at 8 weeks ± 2 weeks. Efficacies were evaluated using the RECIST, and evaluations of efficacies, such as RR, PFS, and OS, can be carried out.	Image evaluations were carried out in clinical practice at the discretion of the attending physician. No evaluations using the RECIST have been conducted. PFS and OS can be evaluated.	Image evaluations were carried out based on standard of care. No evaluations using the RECIST have been conducted. OS, time to therapy discontinuation, RR, and PFS can be evaluated at the patient level.
Evaluation of adverse events	Data of adverse events are stored in the ARCAD database as raw data, and they can be analyzed at the patient level.	Data of adverse events are not collected in principle.	Data of adverse events are not collected in principle.	Data of adverse events are not collected as part of the standard data points, but Flatiron has standard methodologies to extract adverse events, so that rwAE can be analyzed at the patient level, if required.
Informed consents	Consent has been provided by a documented consent form describing secondary use of data. Consents for secondary use, including use by industry, have not been obtained.	Consent has been obtained in a documented consent form describing secondary use, including use by industry.	Consent has been obtained in a documented consent form describing secondary use, including use by industry.	Consent has been obtained in a consent document describing secondary use, such as third-party provision and use by industry.
Legal compliance for regulatory submissions	Under the current Personal Information Protection Law, it may not be possible to use data for regulatory approval.	Data can be used for regulatory approval.	Data can be used for regulatory approval.	Data can be used for regulatory approval.

CRC, colorectal cancer.

## Discussion

By leveraging RWD, Japan’s regulatory bodies have facilitated the development of diverse registries via the CIN. Additionally, the release of several guidelines by the MHLW, coupled with the PMDA’s initiation of consultations for the utilization and integrity assessment of these registries, has progressively solidified the framework for effectively harnessing RWD. On the academic side, registries of superior quality focusing on rare molecular subtypes, rare cancers, and pediatric cancers have been developed. The SCRUM-Japan Registry was used as an external control for the regulatory authorization of pertuzumab plus trastuzumab for HER2-positive, unresectable CRC. This underscores the sophisticated employment of registry data for regulatory objectives when juxtaposed with global benchmarks, signifying the evolving application of registry data within the pharmaceutical domain.

Although the TRIUMPH study represents a notable success, the broader adoption of registry data as external control data in Japan remains limited. This is primarily due to the absence of clear standards for the use of RWD/RWE in drug approval, which has made pharmaceutical companies cautious about leveraging such data in regulatory submissions. In response, we have launched the ‘REALISE study’, aiming to clarify the ‘relevancy’ and ‘reliability’ of the data required for new drug approval and to establish the methodologies for ensuring reliability retrospectively. If sufficient ‘relevance’ and ‘reliability’ of the data are retrospectively obtained from the SCRUM-Japan observational study, well-qualified RWD samples could potentially be utilized at a lower cost. Through the findings of the ‘REALISE study’, we will develop draft guidelines for the application of RWD/RWE to enhance its utilization across Japan. Achieving sustainable registry management will require an ongoing effort to accumulate examples, increase awareness among pharmaceutical companies, and secure continuous support from the MHLW and PMDA. However, the current efforts in Japan to use registries for drug approval have predominantly focused on the expansion of indications. The utilization of RWD for the initial approval of new drugs largely depends on revisions of the International Council for Harmonisation—Good Clinical Practice guidelines.

To improve the utilization of RWD, it is imperative to extract high-quality RWD from EHRs. Commencing in 2021, our exploration into the feasibility of generating high-quality RWD/RWE within Japan has been conducted through a collaborative research initiative with Flatiron Health.^30^ Leveraging the insights derived from this partnership, our objective is to explore methodologies for data extraction that accommodate both structured and unstructured data formats, in addition to examining approaches to ensure data reliability. In Japan, unlike the United States, collaborative research with Flatiron Health is subject to the stringent ‘Act on the Protection of Personal Information’, which necessitates the acquisition of individual consent as a fundamental requirement, and it has a substantial impact on acquisition of consent. Additionally, in Japan, the Act on the Protection of Personal Information does not apply to deceased individuals. Consequently, information is collected based on consent from the patient’s family according to ethical guidelines for research. While treating de-identified data as personal information and handling it with individual consent poses issues such as data bias, complying with the personal data protection regulations of Japan and Europe offers the benefit of creating data that can be utilized in the future without hindrance. To further enhance the use of RWD in clinical research in Japan, it is necessary to discuss and implement more flexible practices to obtain consent in the future.

Furthermore, progress should be made in medical information technology. A significant issue with EHR systems in Japan is the variability in designs among different vendors, coupled with the adoption of designs unique to Japan. To expand the procedures and findings of our study to RWD/RWE from EHRs, standard specifications and medical record terminology for system vendors are required. Next-generation EHRs should be constructed according to the Health Level Seven Fast Healthcare Interoperability Resources to facilitate data integration. Additionally, a certification program for medical information systems, which is common in the United States, should be established. Registering data through appropriate processes using reliable systems has facilitated numerous studies. The data format for this study was constructed based on the Observational Medical Outcomes Partnership Common Data Model, which is the *de facto* international standard format for observational studies.

In conclusion, the use of cancer registry data as an external control has been actively promoted by regulatory bodies and academic institutions. The SCRUM-Japan Registry has played a pivotal role in achieving the first regulatory approval for HER2-positive CRC globally, marking a significant achievement in obtaining regulatory approval through the use of regulatory-grade registries for rare molecular subtypes. To further enhance the utilization of such data, it is imperative to clarify the ‘relevance’ and ‘reliability’ of RWD and RWE, which are necessary for regulatory approval.
